# Determination of Anthraquinone-Tagged Amines Using High-Performance Liquid Chromatography with Online UV Irradiation and Luminol Chemiluminescence Detection

**DOI:** 10.3390/molecules28052146

**Published:** 2023-02-24

**Authors:** Naoya Kishikawa, Mahmoud El-Maghrabey, Ayaka Kawamoto, Kaname Ohyama, Naotaka Kuroda

**Affiliations:** 1 Graduate School of Biomedical Sciences, Course of Pharmaceutical Sciences, Nagasaki University, 1-14 Bunkyo-machi, Nagasaki 852-8521, Japan; 2 Department of Pharmaceutical Analytical Chemistry, Faculty of Pharmacy, Mansoura University, Mansoura 35516, Egypt; 3 School of Pharmaceutical Sciences, Nagasaki University, 1-14 Bunkyo-machi, Nagasaki 852-8521, Japan; 4 Department of Hospital Pharmacy, Nagasaki University Hospital of Medicine and Dentistry, 1-7-1 Sakamoto, Nagasaki 852-8501, Japan

**Keywords:** chemiluminescence, anthraquinone, derivatization, UV irradiation, amine

## Abstract

Quinones are frequently used as derivatization reagents in HPLC analysis to improve detection sensitivity. In the present study, a simple, sensitive, and selective chemiluminescence (CL) derivatization strategy for biogenic amines, prior to their HPLC-CL analysis, was developed. The novel CL derivatization strategy was established based on using anthraquinone-2-carbonyl chloride as derivatizing agent for amines and then using the unique property of the quinones’ moiety to generate reactive oxygen species (ROS) in response to UV irradiation. Typical amines such as tryptamine and phenethylamine were derivatized with anthraquinone-2-carbonyl chloride and then injected into an HPLC system equipped with an online photoreactor. The anthraquinone-tagged amines are separated and then UV-irradiated when they pass through a photoreactor to generate ROS from the quinone moiety of the derivative. Tryptamine and phenethylamine can be determined by measuring the chemiluminescence intensity produced by the reaction of the generated ROS with luminol. The chemiluminescence disappears when the photoreactor is turned off, suggesting that ROS are no longer generated from the quinone moiety in the absence of UV irradiation. This result indicates that the generation of ROS could be controlled by turning the photoreactor on and off. Under the optimized conditions, the limits of detection for tryptamine and phenethylamine were 124 and 84 nM, respectively. The developed method is successfully applied to determine the concentrations of tryptamine and phenethylamine in wine samples.

## 1. Introduction

In order to investigate the benefits of a food or natural product, it is essential to analyze its components [1,2,3]. Among analytical techniques, high-performance liquid chromatography (HPLC) is a particularly powerful tool because it allows the simultaneous determination of concentrations of many components. In HPLC, each component after separation in a column is detected based on several detection methods, including ultraviolet (UV) detection, fluorescence detection, electrochemical detection, and mass spectrometry (MS). In this case, derivatization, a method of binding a signal amplification tag to the target analyte, is often used to improve detection sensitivity [4,5]. There are various types of signal amplification tags, including chromophores, fluorophores, electroactive species, and ionizable moieties [6,7]. Quinone is one such signal amplification tag with unique versatility. For example, 1,2-naphthoquinone-4-sulfonate (Folin’s reagent) has been widely adopted for the introduction of the chromophore to amines [8,9,10]. Anthraquinone acid chloride, including anthraquinone-2-carbonyl chloride and anthraquinone-2-sulfonyl chloride, are used as derivatization reagents for HPLC-UV and LC-MS of amines and phenols as they form colored and ionizable derivatives through amidation and esterification reactions [11,12,13]. *ortho*-Quinone has been reported to react with aldehydes to give fluorescent and ionizable imidazole derivatives [14,15], and these techniques have been applied to the HPLC analysis of aldehydes such as nonenal in biological samples. In addition, our research group has developed novel methods to use quinones as reactive oxygen species (ROS) generation tags for sensitive chemiluminescence (CL) detection based on the redox reaction of quinone [16,17,18,19]. In these methods, (1) quinones are reduced by dithiothreitol (DTT) to yield unstable semiquinone radicals; (2) semiquinone radicals convert dissolved oxygen to ROS and themselves are oxidized to the original quinone; (3) ROS reacts with luminol to produce CL. It was possible to determine quinone by measuring the CL produced through the above reactions. As an application to HPLC analysis, we reported a determination method of aminothiols in biological samples using menadione as a pre-column derivatization reagent [20,21]. The Michael addition reaction between menadione and aminothiol yielded a menadione-tagged aminothiol, which retained the ROS-generating capability of the parent menadione. The menadione-tagged aminothiols, after chromatographic separation, were mixed with DTT, and the generated ROS was detected by luminol CL reaction.

Interestingly, we found that quinones have excellent photosensitization properties and that their photoirradiation generates ROS [22,23]. Based on this finding, we have developed several luminol CL determination methods for quinone using HPLC systems equipped with an online photoreactor [24,25]. Therefore, we considered that the CL detection of quinone-tagged compounds could be achieved by photoirradiation instead of the addition of DTT. First, the analyte is derivatized with a quinone reagent that generates ROS upon photoirradiation. Next, the quinone-tagged analytes, after separation, are introduced into the online photoreactor to generate a ROS, which is then mixed with a luminol solution, and the resulting CL is monitored. In the present study, we attempted to develop a determination method for biogenic amines by HPLC with online UV irradiation and CL detection.

Biogenic amines are bio-nitrogenous compounds that mainly result from the enzymatic decarboxylation of amino acids that have very important biological functions, including stabilization of the cell membrane, immunity boosting, and providing protection against many chronic diseases, owing to their partaking in the synthesis of very important nucleic acids and proteins [26]. On the other hand, biogenic amines are present in many foods, either raw or processed, including vegetables, cheese, and beverages. From a microbiological point of view, biogenic amines found in large amounts in foods are occasionally associated with spoilage and fermentation [27]. Up to now, there have been many reported chromatographic and capillary electrophoresis methods for the determination of biogenic amines in food and biological samples after their precolumn derivatization, followed by UV, fluorescence (FL), and mass spectrometric detection [28,29,30,31,32,33]. Prior to their HPLC analysis, biogenic amines are mostly derivatized to enhance their chromatographic separation and eliminate the matrix effect, and for increasing detection sensitivity [34]. The most commonly used derivatizing agents for biogenic amines are benzoyl chloride [28], dansyl chloride [30], *o*-phthalaldehyde [35], and fluorenyl methyl chloroformate [36]. All of these derivatizing agents enable the UV, FL, or MS detection of biogenic amines.

On the other hand, CL detection possesses inherent sensitivity due to the absence of background light, inherent selectivity because of the scarcity of the CL reactions, and cost-effectiveness due to the low cost and simplicity of the CL detector. In addition, as CL is generated from a chemical reaction and does not need an excitation source, CL is not affected by external factors such as stray and scattered light that largely affect fluorescence detection which needs an excitation source [37,38]. Our research group is concerned with developing new CL derivatizing reagents for compounds that have rarely reported CL detection methods. The dinitrophenyl hydrazine (DNPH) derivatized aldehydes were assayed by HPLC-CL based on their photosensitization for the first time by our research group. The UV-irradiated DNPH-aldehydes were found to form ROS, including singlet oxygen and hydroperoxide, which react online with luminol forming strong CL. This method was the first chemiluminogenic method for aldehydes [37].

**Figure 1:**
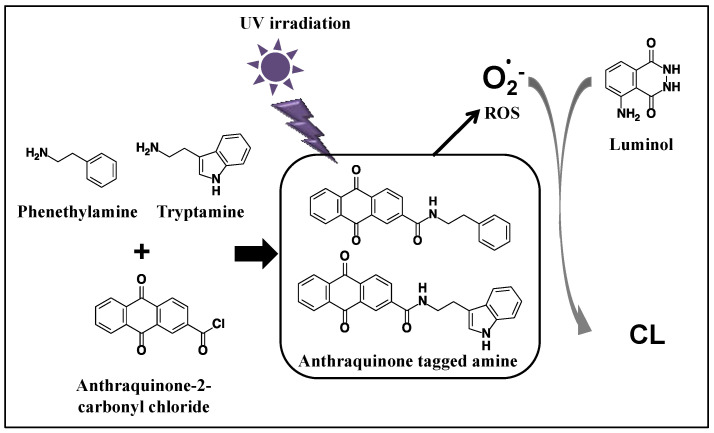
Derivatization of tryptamine and phenethylamine with anthraquinone-2-carbonyl chloride and luminol CL reaction after UV irradiation.

Despite the described excellent advantages of CL detection, until now, no selective chemiluminogenic reagents for biogenic amines have been developed. Therefore, herein, we report a new CL derivatization strategy for the HPLC-CL determination of typical amines through derivatization with anthraquinone-2-carbonyl chloride, as seen in Figure 1. The typical biogenic amines tryptamine and phenethylamine were derivatized with anthraquinone-2-carbonyl chloride and then injected into an HPLC-CL system equipped with an online photoreactor. The anthraquinone-tagged amines are separated, and then UV-irradiated in the photoreactor, and consequently, ROS are generated. Next, chemiluminescence is produced by the online reaction of the generated ROS with luminol. Finally, the developed method was applied to the determination of amines in wine samples.

## 2. Result and Discussion

### 2.1. Chromatogram of Amines after the Reaction with Anthraquinone-2-Carbonyl Chloride

First, the anthraquinone derivatives of tryptamine and phenethylamine were separated on an ODS column using a mixture of acetonitrile and imidazole trifluoroacetic acid buffer, pH 7. Increasing the acetonitrile% than 60 % lead to poor separation of the biogenic amines derivative, while decreasing acetonitrile% than 60 % lead to slightly broadened peaks. Hence, the optimum ratio of acetonitrile to the aqueous buffer was 60/40 (*v/v*, %). The separation was conducted successfully at room temperature (23–27 °C). The effect of buffer pH and strength was found to be nearly negligible on the retention times of the biogenic amines’ derivatives. Hence, the buffer concentration was studied only for enhancing CL detection, as discussed later in Section 2.3.

**Figure 2:**
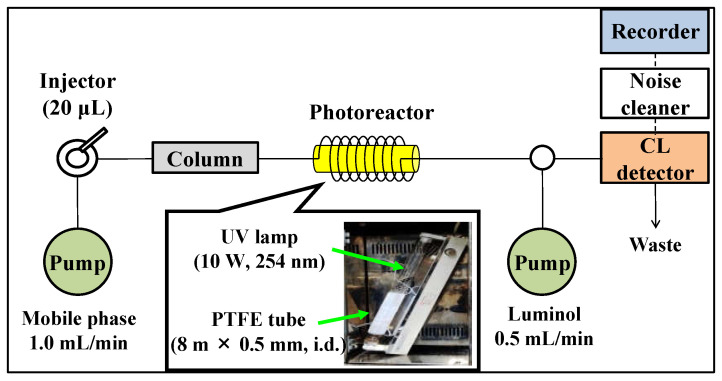
Scheme diagram for the HPLC with online photoreactor and luminol CL detection system.

**Figure 3:**
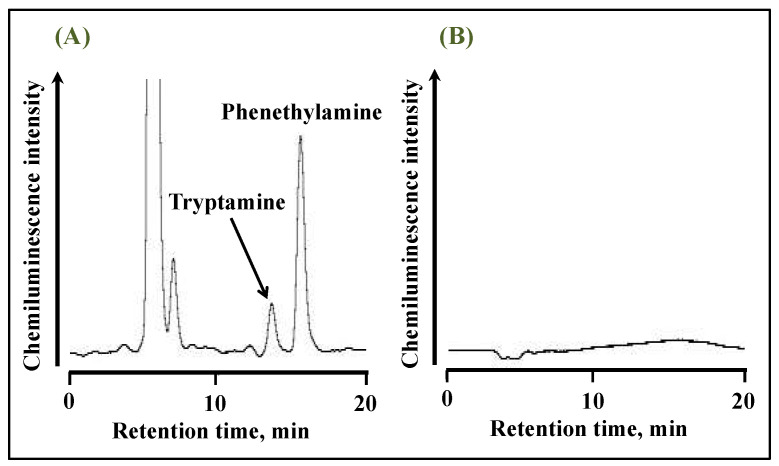
Chromatograms of 10 µM standard solution of tryptamine and phenethylamine after derivatization with anthraquinone-2-carbonyl chloride obtained (**A**) with and (**B**) without UV irradiation.

A schematic diagram of the HPLC with an online UV irradiation and CL detection system is shown in Figure 2. The eluate from the separation column is UV-irradiated when it passes through a tube coiled around to a low-pressure mercury lamp and then mixed with a luminol solution and introduced into the CL detector. Figure 3 shows typical chromatograms of a standard solution of tryptamine and phenethylamine after derivatization with anthraquinone-2-carbonyl chloride. The derivatives of tryptamine and phenethylamine were detected at a retention time of 13 and 15 min, respectively, under UV irradiation (Figure 3A). In addition, peaks derived from the derivatization reagent anthraquinone-2-carbonyl chloride and its degradation product anthraquinone were detected at retention times of 6 and 7 min, respectively, while these peaks disappeared when UV irradiation was turned off (Figure 3B). It is suggested that CL is produced by the reaction of ROS generated from the anthraquinone-tagged amine with luminol. Since the ROS required for luminol CL could be supplied from the quinone tagged to the target analyte, the preparation and delivery of the ROS reagent could be omitted. In addition, CL is initiated by the generation of ROS only upon UV irradiation of the anthraquinone-tagged compounds. Therefore, CL can be produced at the appropriate timing, enabling efficient CL measurement.

As mentioned previously, the CL produced from the UV irradiation of the reaction product of biogenic amines with anthraquinone-2-carbonyl chloride after its mixing with luminol is due to the quinone content of the reaction product. Previously, our research group had developed the first luminol CL detection of quinones after their UV irradiation. The mechanism of such photochemically derived luminol CL for quinones was thoroughly studied in our previous publication [25]. The mechanism could be summarized as follows. Upon UV irradiation of quinones or their derivatives, two main products are produced; the first one is ROS (mainly superoxide anion radical), while the second one is 3,6-dihydroxy phthalic acid (DHPA) [22]. Then, DHPA is autoxidized into semiquinone radical, producing more superoxide anion radicals. Next, the semiquinone radical reacts with luminol anion, producing luminol anion radical. At this stage, the previously produced superoxide anion radical reacts with the luminol anion radical, producing the excited state of 3-aminophthalate that then emits light (CL) while returning to its ground state [25].

### 2.2. Optimization of Derivatization Reaction

**Figure 4:**
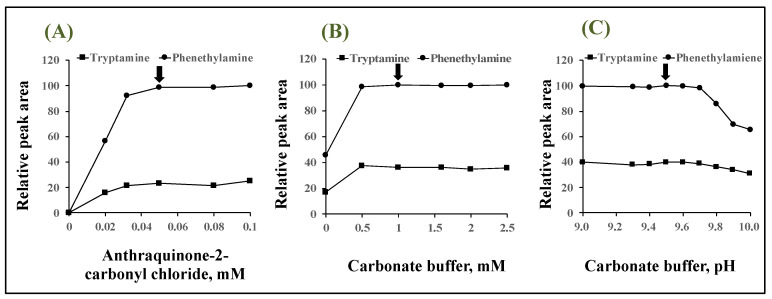
Effects of (**A**) anthraquinone-2-carbonyl chloride concentration, (**B**) carbonate buffer concentration, and (**C**) buffer pH on the peak area of derivative. Arrows indicate optimum conditions.

In order to obtain higher reactivity, derivatization conditions were optimized. The relative peak areas were calculated related to the area of the phenethylamine derivative, with the highest value among tested conditions for phenethylamine as 100. Concentrations of anthraquinone-2-carbonyl chloride were investigated in the range of 0–0.1 mM (Figure 4A). As the reagent concentration increased, the CL increased until it reached a plateau at 0.05 mM. As a result, 0.05 mM was selected as the anthraquinone-2-carbonyl chloride concentration because it yielded the largest peak area with minimum reagent consumption. The effect of base type on the reaction was investigated using 1.0 mM carbonate buffer (pH 9.5), 1.0 mM triethylamine aqueous solution, 1.0 mM pyridine aqueous solution, and 1.0 mM sodium hydroxide aqueous solution as a base catalyst. Among the bases examined, carbonate buffer (pH 9.5) gave the largest peak area and thus was selected. The effect of the concentration of the carbonate buffer was investigated in the concentration range of 0–2.5 mM. As seen in Figure 4B, the concentration of the buffer in the range of 0.5–2.5 mM yielded nearly the same CL; hence 1.0 mM carbonate buffer was used in further studies (Figure 4B). Subsequently, optimization of the pH of the 1.0 mM carbonate buffer solution resulted in an almost maximum and constant peak area in the pH range 9.3–9.7 (Figure 4C). Therefore, 1.0 mM carbonate buffer (pH 9.5) was selected as the base catalyst.

The effect of reaction temperature and time on the derivatization reaction was investigated. Since there was no change in peak area with increasing reaction temperature, the derivatization reaction was carried out at room temperature (approximately 23 to 27 °C). Optimization of the reaction time at room temperature showed that the peak area did not increase with increasing reaction time, suggesting that the reaction was completed quickly after the addition of anthraquinone-2-carbonyl chloride. Therefore, room temperature and 10 s were selected for the reaction temperature and time, respectively.

### 2.3. Optimization of UV Irradiation and Chemiluminescence Conditions

**Figure 5:**
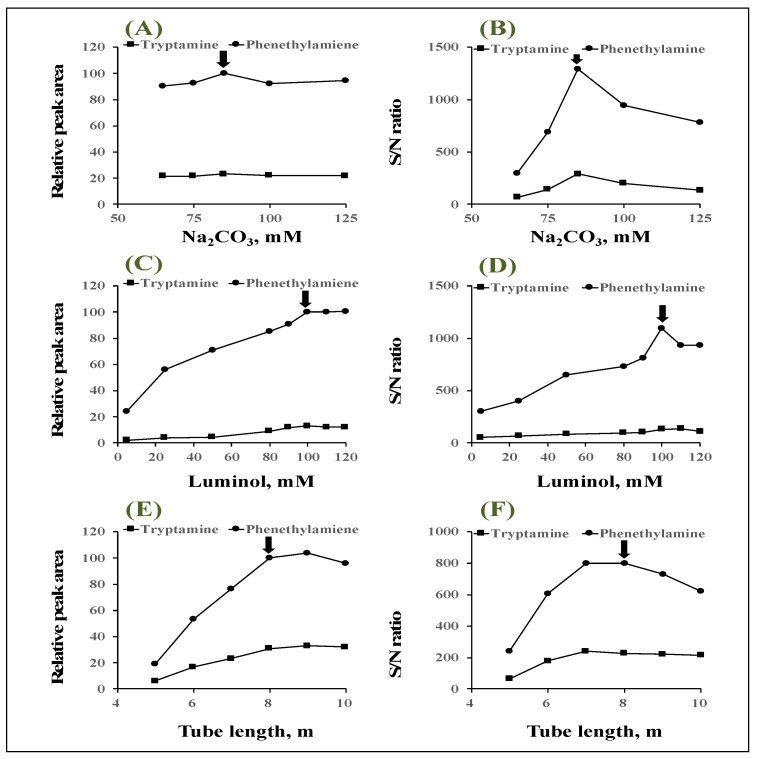
Effects of sodium carbonate concentration on (**A**) relative peak area and (**B**) S/N ratio. Effects of luminol concentration on (**C**) relative peak area and (**D**) S/N ratio. Effects of tube length of photoreactor on (**E**) relative peak area and (**F**) S/N ratio.

In order to obtain higher CL intensity, UV irradiation and luminol CL conditions were optimized. The effect of base solvent to dissolve luminol was investigated using 100 mM sodium carbonate aqueous solution, 100 mM sodium hydroxide aqueous solution, 100 mM carbonate buffer (pH 9.5, 10.5, and 12.0), 100 mM borate buffer (pH 10.5), and 100 mM phosphate buffer (pH 12.0). Among the base solvents tested, sodium hydroxide gave the largest peak area but also increased the noise, while the highest S/N ratio was obtained using sodium carbonate. Therefore, sodium carbonate aqueous solution was used as a solvent for luminol. Concentrations of sodium carbonate solution were investigated in the range of 65–125 mM. As shown in Figure 5A, the peak area of both tryptamine and phenethylamine reached a maximum of 85 mM, then the peak area became nearly constant, while in Figure 5B, the S/N ratio of both tryptamine and phenethylamine reached a maximum at only 85 mM, so 85 mM was selected as the optimum concentration.

Next, the effect of luminol concentration on the peak area and S/N ratio was investigated (Figure 5C,D). The peak area increased with increasing luminol concentration, reaching a maximum and constant of more than 0.1 mM (Figure 5C). On the other hand, background noise increased with luminol concentration above 0.1 mM, at which concentration, the S/N ratio reached a maximum (Figure 5D). Therefore, the optimum luminol concentration was selected at 0.1 mM. This increase in noise might be attributed to the spontaneous degradation of high concentrations of luminol. The flow rate of 0.1 mM luminol solution was optimized in the range of 0.3–0.7 mL/min. The peak areas and S/N ratios for both tryptamine and phenethylamine were maximum at 0.5 mL/min, so the flow rate of the luminol solution was set at 0.5 mL/min. As imidazole was reported as a catalyst for quinones’ photodegradation into DHPA owing to its strong nucleophilic catalytic properties, it was used as a buffer in combination with trifluoroacetic acid [22,25]. The optimal concentration of imidazole in the imidazole–trifluoroacetic acid buffer, used as the mobile phase, was investigated in the range of 50–120 μM after adjusting the pH to 7.0. As a result, the peak area and S/N ratio were maximum when the imidazole concentration was 80 mM. Therefore, 80 mM was selected as the best imidazole concentration. The proposed HPLC system is based on the generation of ROS from anthraquinone-tagged amine by UV irradiation to the eluate from the column. The UV irradiation time could be controlled by the length of the tube coiled around the lamp. Therefore, the length of the UV-irradiation tube has a significant influence on the CL reaction. The optimization of the tube length was investigated in the range of 5–10 m. Although the peak area increases with increasing tube length (Figure 5E), the best S/N ratio was obtained at a tube length of 8 m because of a concomitant increase in noise (Figure 5F). Therefore, 8 m was selected as the optimum tube length.

Next, as light sources for the photoreactor, lamps with wavelengths of 254 nm and 352 nm were available. The UV lamp of 254 nm was employed because the maximum absorption wavelength of anthraquinone-2-carbonyl chloride was 258 nm. It was thought that by increasing the output of UV irradiation, the efficiency of ROS generation would increase, and higher sensitivity could be achieved. Therefore, CL measurement was carried out by a photoreactor equipped with a 15 W UV lamp (254 nm). As expected, the peak area increased compared to the 10 W UV lamp, but at the same time, the noise level also increased, and the S/N ratio was not improved. Thus, the UV lamp (10 W, 254 nm) was used to construct the photoreactor.

### 2.4. Calibration Curve, the Limit of Detection, and Precision Study

Under the optimum conditions, calibration curves were constructed using the standard solutions of tryptamine and phenethylamine. The calibration curves, calibration ranges, and limits of detection (LOD) are listed in Table 1. The calibration curve was constructed using seven different concentrations of the targeted analytes. The calibration curve for tryptamine was linear over the range of 0.2–20 μM, with a correlation coefficient of 0.998, while that for phenethylamine was linear over the range of 0.1–10 μM with a correlation coefficient of 0.999. The LODs of tryptamine and phenethylamine, calculated at a signal-to-noise ratio of 3, were 124 and 84 nM, respectively.

The sensitivity of the proposed method was compared with some of the previously reported methods for the determination of biogenic amines in urine and food samples and as summarized in Table 2. The developed HPLC-CL method was 10–49 times more sensitive than the HPLC-UV method [28], 2.5–7 times sensitive than the capillary zone electrophoresis (CZE) method [29], 5–10 times sensitive than the HPLC-FL method [30], and had comparable sensitivity to LC with quadrupole time-of-flight tandem mass spectrometry [31] and the HPLC-UV method developed by Jia et al. [32], who used sophisticated in situ benzoylation assisted with dispersive liquid–liquid microextraction. On the other hand, the proposed method was less sensitive when compared to the LC with tandem mass spectrometry, but the proposed HPLC system has much lower equipment and running costs than the LC-MS/MS system [33].

The precision of the proposed method was evaluated using the three concentrations of tryptamine and phenethylamine within the calibration range. The intra-day precision was measured by analyzing the concentrations 0.5, 2.5, and 10.0 µM five times on the same day, while the inter-day precision was measured by analyzing these concentrations on five successive days. As shown in Table 3, the relative standard deviation (RSD) in the intra-and inter-day were less than 6.1 and 9.8% for tryptamine, and 5.7 and 9.9% for phenethylamine, respectively. These results indicate the good precision of the proposed method (Table 3).

### 2.5. Application for the Determination of Amines in Wine Samples

Biogenic amines were previously reported to be present in many wine samples [34]. As is well known, the primary precursors of biogenic amines in wine are amino acids [39]. Additionally, biogenic amines could be produced in wine during alcoholic fermentation. Furthermore, amino acids could undergo decarboxylation by the effect of many yeasts and bacteria originating from spoilage, yielding biogenic amines. Moreover, certain carbonyl compounds, including aldehydes and ketones, could undergo fermentation and aging-induced amination and/or transamination, producing biogenic amines [40]. There are various influences that affect biogenic amine levels in wine. Among these factors, the grape type, cultivated climate, the manufacturing process, and aging are the most significant [34]. Among the main biogenic amines that are present in wine, tryptamine, and phenylethylamine were reported [41]. Hence, the developed method was applied to the determination of tryptamine and phenethylamine in red and white wine samples. Monitoring of tryptamine and phenethylamine in fermented foods such as wine and cheese is necessary because excessive consumption of these amines can cause headaches and elevated blood pressure [33]. In order to extract tryptamine and phenethylamine, the method of Francisco et al. [42] was adopted. The samples were alkalinized with NaOH, then the salting-out liquid–liquid extraction (SALLE) technique was induced using NaCl. The recoveries of tryptamine and phenethylamine from wine samples were studied by spiking pre-analyzed wine samples with the targeted analytes. Then, using SALLE, the targeted analytes were extracted, followed by derivatization with anthraquinone-2-carbonyl chloride and HPLC-CL analysis. The following rule was used to calculate % recovery.

% Recovery = Found concentration/(spiked concentration − original concentration) × 100

**Figure 6:**
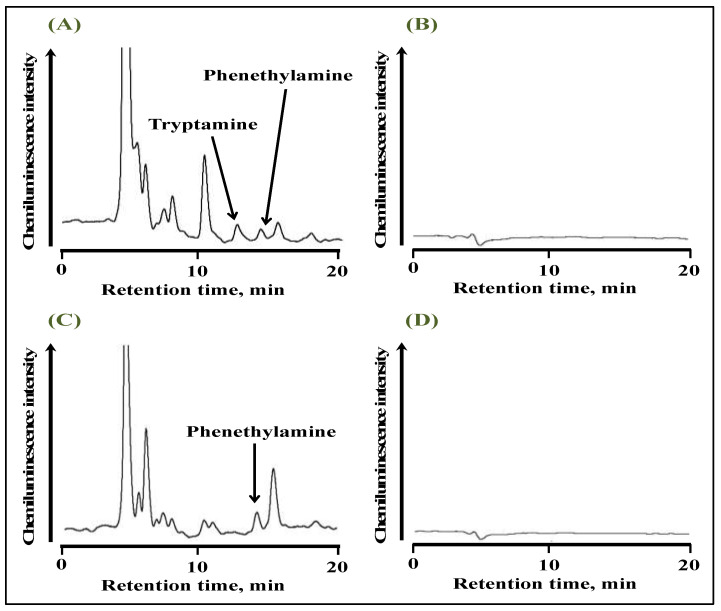
Chromatograms of red wine obtained (**A**) with and (**B**) without UV irradiation, and white wine obtained with (**C**) with and (**D**) without UV irradiation.

The % recoveries were found to be 92 and 88 % for tryptamine and phenethylamine, respectively. Red and white wines were extracted using SALLE prior to their derivatization with anthraquinone-2-carbonyl chloride, and the typical chromatograms are shown in Figure 6. Both tryptamine and phenethylamine were detected in red wine (Figure 6A), while only phenethylamine was detected in white wine (Figure 6C). The peaks detected on the chromatogram, including tryptamine and phenethylamine, disappeared in the absence of UV irradiation (Figure 6B,D). Therefore, these peaks could be attributed to wine components derivatized with anthraquinone-2-carbonyl chloride. The concentrations of tryptamine and phenethylamine in wine samples quantified by the standard addition method are summarized in Table 4, and these values were in good agreement with those of previously reported studies [32,33].

## 3. Experimental Section

### 3.1. Material and Reagents

Anthraquinone-2-carbonyl chloride and imidazole were purchased from Tokyo Chemical Industry (Tokyo, Japan). Acetonitrile was obtained from Kanto Chemical (Tokyo). Luminol, sodium hydrogen carbonate, and sodium hydrogen carbonate were obtained from Nacalai Tesque (Kyoto, Japan). Tryptamine, sodium hydroxide, and trifluoroacetic acid were obtained from Wako Pure Chem. Co. (Osaka, Japan). Phenethylamine hydrochloride was purchased from Sigma (St. Louis, MO, USA). Purified water was generated using Autostill WG 203 (Yamato Scientific Co., Ltd., Tokyo, Japan). Other chemicals were of extra-pure grade. Wine samples were purchased from local markets in Nagasaki city. Anthraquinone-2-carbonyl chloride was dissolved in acetonitrile. Stock solutions of tryptamine and phenethylamine were prepared with water and ethanol, respectively, and diluted with acetonitrile to prepare working solutions.

### 3.2. HPLC System and Conditions

The HPLC system (Figure 2) consisted of two LC-10AS liquid chromatographic pumps (Shimadzu, Kyoto, Japan), a Rheodyne 7125 injector (Cotati, CA, USA) with a 20-μL sample loop, a CLD-10A CL detector (Shimadzu), a UNI-1 noise filter (Union, Gunma, Japan), a GL-10 UV lamp (10 W, 254 nm, Hitachi, Tokyo, Japan), and a Chromato-Pro data acquisition system (Run-time Instruments Co., Ltd., Tokyo, Japan). The injection volume of the reaction mixture into the HPLC system was 20 µL. The chromatographic separation was performed at room temperature (approximately 23 to 27 °C) on a Daisopak SP-120-5-ODS-BP (250 × 4.6 mm, i.d., 5 μm, Daiso, Osaka, Japan) using a mobile phase consisting of acetonitrile and 80 mM imidazole-trifluoroacetic acid buffer (pH 7.0) in the ratio of 60/40 (*v/v*, %). PTFE tubing (8 m × 0.5 mm i.d., GL Sciences, Tokyo, Japan) coiled around the UV lamp was used as the online photoreactor. Anthraquinone-tagged amine was UV-irradiated while passing through the tubing, then mixed with 85 mM NaOH solution of 100 µM luminol and introduced into the CL detector. The flow rates of the mobile phase and luminol solution were set at 1.0 and 0.5 mL/min, respectively.

### 3.3. Derivatization of Amines with Anthraquinone-2-Carbonyl Chloride

Aliquots of 50 μL tryptamine and phenethylamine were mixed with 100 μL of 0.05 mM anthraquinone-2-carbonyl chloride and 50 µL of carbonate buffer (pH 9.5). The reaction mixtures were vortex-mixed at room temperature for 10 s. Then, 20 μL of the reaction solution was injected into the HPLC system.

### 3.4. Assay Procedure for Amines in Wine Samples

The extraction of tryptamine and phenethylamine from wine samples was accomplished by salting-out liquid–liquid extraction. To 100 µL of wine sample, 400 µL of 0.1 M sodium hydroxide, 500 µL of acetonitrile, and 0.2 g sodium chloride were added. After vortex mixing for 30 s, 50 µL of the supernatant was collected after centrifugation at 500 g for 5 min. The supernatant was then subjected to derivatization with anthraquinone-2-carbonyl chloride.

## 4. Conclusions

In this study, we developed a novel CL derivatization strategy for typical amines, including tryptamine and phenethylamine, with the anthraquinone-2-carbonyl chloride based on the ROS-generating capability of quinone. UV irradiation provides the generation of ROS from the anthraquinone tagged to the amine, and the CL produced by the reaction of the generated ROS with luminol allows the measurement of amines. The proposed method, since ROS is generated from the target derivative, does not require pumping ROS solution like general HPLC with a luminol CL system. Thereby, simplifying the system and improving reproducibility are achieved. In addition, the reaction between amines and anthraquinone-2-carbonyl chloride proceeds within 10 s, demonstrating the speed of the derivatization protocol. In order to demonstrate the practicability, the proposed method was successfully applied to the determination of tryptamine and phenethylamine in wine samples after their extraction using a simple SALLE method. The proposed method would be useful for monitoring various amines that contribute to the benefits of foods and natural products.

## Figures and Tables

**Table 1:** Calibration curve and LOD of anthraquinone tagged amine.

	Linear Range, μM	Calibration Equation *	Correlation Coefficient (r)	LOD **, nM
Tryptamine	0.2–20	Y = 2.8 × 10^5^ X + 8.6 × 10^3^	0.998	124
Phenethylamine	0.1–10	Y = 1.1 × 10^6^ X + 1.2 × 10^5^	0.999	84

* Y = Peak area; X = Concentration of amine, μM ** Limit of detection, S/N = 3.

**Table 2:** Calibration curve and LOD of anthraquinone-tagged amine.

Method	Derivatizing Agent	LOD		Sample	Ref
		Tryptamine	Phenethylamine		
HPLC-UV	Benzoyl chloride	1248	4126	Wine samples	[28]
CZE-UV	None	300	600	Urine	[29]
HPLC-FL	Dansyl chloride	625	825	Wine	[30]
LC-MS/MS	Dansyl chloride	125	41	Food samples	[31]
LC-MS/MS	4’-Carbonyl chloride rosamine	2.5	2.5	Food samples	[32]
HPLC-UV	Benzoyl chloride	63	83	Wine	[33]
HPLC-CL	Anthraquinone-2-carbonyl chloride	124	84	Wine	This work

**Table 3:** The results of the reproducibility study of the proposed method.

	Concentration, µM	Precision (RSD, %)	
		Intra-Day (n = 5)	Inter-Day (n = 5)
Tryptamine	0.5	3.3	9.4
	2.5	4.7	7.4
	10	6.1	9.8
Phenethylamine	0.2	4.3	8.5
	2.5	5.7	6.3
	10	5.2	9.9

**Table 4:** The found concentration of amines in wine samples.

Sample	Concentration, µM	
	Tryptamine	Phenethylamine
Red wine 1	1.7	2.3
Red wine 2	3.3	4.2
White wine 1	n.d.*	4.6
White wine 2	n.d.*	1.3

* Not detected.

## Data Availability

The data will be available upon reasonable request.

## Sample Availability

Samples of the compounds are not available from the authors.
